# Lady Churchill and Mrs. Maybrick

**Published:** 1900-01

**Authors:** 


LADY CHURCHILL AND MRS. MAYBRICK. In our "Abstracts and Selections" we reproduce from advance sheets *Medico-Legal Journal*, a letter to Lady Churchill from members of the Psychological Section of the Medico-Legal Society, to which we call attention. An appeal has been made to America for contributions to help fit out a hospital ship for British wounded in Africa, and the members of the Psychological Section take the occasion to make a counter-appeal to Lady Churchill to use her influence in securing the release of Mrs. Maybrick who is, and for ten years has been, suffering an unjust punishment in an English prison. Read the letter; it will explain itself. The continued incarceration of this American lady, and the persistent refusal of the English authorities to heed the protests of the American people, especially under existing circumstances, is more infamous than the punishment of Dreyfus. Until justice is done her, and some respect shown the request of the President of the United States to at least look into the matter,—a request based upon the unanimous opinion of all who are acquainted with the facts,—not the least amongst whom are the Lord Chief Justice of England and the trial judge, not a cent of American money should be sent to help even the wounded, although it is an appeal in the name of humanity. Ours is not only an appeal to humanity, but for justice, and the good old queen, who was so tender hearted as to weep at the news of the slaughter of her soldiers in the Boer war, seems deaf to the wrongs and suffering of an American lady who had the misfortune to marry a worthless Englisman.

Judge Clark Bell, the distinguished President of the Medico-Legal Society of America, the brilliant advocate of New York, and editor of the *Medico-Legal Journal,* has been indefatigable in his efforts to secure Mrs. Maybrick's release. The letter referred to was doubtless drafted by him, and when Mrs. Maybrick is finally released, as she must be, or the United States should make the case a *casus belli,* the American people will have him mainly to thank for it.

